# Infant and young child feeding indicators are positively associated with length and family care indicators in the children of the Women First trial participants

**DOI:** 10.1111/mcn.13572

**Published:** 2023-10-10

**Authors:** Julie M. Long, Giovanna Gatica‐Domínguez, Jamie E. Westcott, Douglas Taren, Gabriela Tejeda, Tshilenge S. Diba, Shivanand C. Mastiholi, Umber S. Khan, Ana Garcés, Lester Figueroa, Adrien Lokangaka, Shivaprasad S. Goudar, Sumera Aziz Ali, K. Michael Hambidge, Nancy F. Krebs, the Women First Preconception Maternal Nutrition Study Group

**Affiliations:** ^1^ Department of Pediatrics, Section of Nutrition University of Colorado School of Medicine Aurora Colorado USA; ^2^ Maternal and Infant Health Center Instituto de Nutrición de Centro América y Panamá Guatemala City Guatemala; ^3^ Kinshasa School of Public Health, Democratic Republic of Congo Kinshasa Democratic Republic of the Congo; ^4^ Women's and Children's Health Research Unit KLE Academy of Higher Education and Research's Jawaharlal Nehru Medical College Belagavi India; ^5^ Department of Community Health Sciences Aga Khan University Karachi Pakistan

**Keywords:** child feeding, complementary feeding, Democratic Republic of Congo, growth, Guatemala, India, low‐ and middle‐income countries, nurturing care framework, Pakistan

## Abstract

This research describes the proportion of children in four low‐ and middle‐income countries with adequate dietary practices at 6, 12, 18 and 24 months of age and how these practices changed over time using the World Health Organisation and UNICEF's infant young child feeding (IYCF) indicators. The associations between the IYCF indicators and anthropometric z‐scores from 6 to 24 months, and between the IYCF indicators and the family care indicators (FCIs) at 24 months are described. This was a longitudinal study of offspring from participants in the Women First Preconception Maternal Nutrition Trial conducted in Sud‐Ubangi, Democratic Republic of Congo; Chimaltenango, Guatemala; Belagavi, North Karnataka, India; and Thatta, Sindh Province, Pakistan. The frequency of the minimum dietary diversity (MDD), minimum meal frequency (MMF), and minimum adequate diet (MAD) increased between 6 and 24 months, but even at 24 months MAD remained below 50% at all sites. MDD (*β* = 0.12; 95% CI = 0.04−0.22) and MMF (*β* = 0.10; 95% CI = 0.03−0.17) were positively associated with length‐for‐age z‐score at 24 months. All IYCF indicators were positively associated with mean total FCI score: MDD (proportion ratio [PR] = 1.04; 95% CI = 1.02−1.07), MMF (PR = 1.02; 95% CI = 1.01−1.04), MAD (PR = 1.05; 95% CI = 1.02−1.08). Although there are multiple barriers to young children having an adequate diet, our results support a positive association between familial interactions and improved IYCF feeding practices.

## Key messages

- Minimum dietary diversity (MDD), minimum meal frequency (MMF) and minimum adequate diet (MAD) increased from 6 to 24 months of age, but MAD remained below 50% frequency at all sites at 24 months.
- MDD and MMF were positively associated with length‐for‐age z‐scores at 24 months.
- The total family care indicator score was positively associated with all three infant young child feeding indicators at 24 months.

## Graphical abstract

The infant young child feeding (IYCF) indicators increased from 6 to 24 months in young children in rural settings of four low‐ and middle‐income countries: Democratic Republic of the Congo, Guatemala, India and Pakistan, but the percentage of children who met the minimum dietary diversity (MDD) and minimum acceptable diet remained below 50% at 24 months. MDD and minimum meal frequency were positively associated with length‐for‐age z‐scores at 24 months (solid lines), and the total family care indicator score was positively associated with all three IYCF indicators at 24 months (dashed lines).

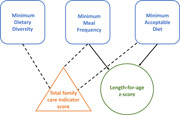

## 1 INTRODUCTION

Child undernutrition is a complex problem with potential consequences, including poor growth and decreased cognitive and motor development (Black et al., 2013). Substandard complementary feeding practices, including heavy reliance on staple crops and limited intake of animal source foods (ASF) and fruits and vegetables are risk factors for suboptimal childhood health, growth and development (Baye & Kennedy, 2020; Black et al., 2013; White et al., 2017). From about 6 months of age onward, breast milk alone is not sufficient to meet all nutritional requirements, and simultaneously by 6 months, infants' motor development enables safe advancement to semi‐solid and eventually solid foods. From 6 to 24 months of age, the risk of malnutrition, including micronutrient deficiencies, increases if adequate complementary feeding practices are not implemented. Observational studies have demonstrated that better maternal dietary diversity, greater child diet diversity, increased frequency of daily meals and consumption of ASF and micronutrient‐fortified foods are associated with better child nutritional status and growth (Arimond & Ruel, 2004; Bonis‐Profumo et al., 2021; Hanley‐Cook et al., 2020; Hasan et al., 2019; Krebs et al., 2011).

To better assess complementary feeding practices, UNICEF and the World Health Organisation (WHO)'s Technical Expert Advisory Group on Nutrition Monitoring (TEAM) developed the infant young child feeding (IYCF) indicators in 2008, (WHO, 2010) updated in 2018 (WHO and United Nations Children's Fund UNICEF, 2021). Three of the core indicators evaluate complementary feeding practices from 6 to 24 months of age, including minimum dietary diversity (MDD), minimum meal frequency (MMF) and minimum acceptable diet (MAD). These indicators were chosen because better dietary diversity and frequent meal consumption are associated with energy and micronutrient intakes as well as better growth (WHO and United Nations Children's Fund UNICEF, 2021). The IYCF indicators provide a standardised tool to qualitatively assess current infant and young child feeding practices, including trends over time, identifying populations at risk and monitoring the progress and improvement of those at‐risk populations.

The family care indicator (FCI) tool, developed by UNICEF in 2002, aims to characterise the family's involvement and structural stimulation of children during their early years of life (Hamadani et al., 2010). Positive familial engagement in the early years improves the cognitive, emotional and motor skills of a child, which has long lasting positive effects throughout life (Jeong et al., 2017). The FCI has successfully been implemented in low‐ and middle‐income countries (LMICs) with the potential to expand its use (Hamadani et al., 2010).

Higher FCI scores have been associated with better cognitive development in young children (Rubio‐Codina & Grantham‐McGregor, 2020; Zhong & Luo, 2020). Responsive feeding offers an important opportunity for young children to interact with their caregivers and improve their nutritional status (Black & Aboud, 2011). Higher FCI scores and responsive feeding practices have been associated with more responsive parenting. One systematic review found that responsive feeding practices improved children's nutrient intake and complementary feeding practices (Bentley et al., 2011). Two components of the Nurturing Care Framework for Early Development include adequate nutrition and opportunities for early learning (United Nations Children's Fund UNICEF and WHO, 2022). The IYCF indicators are a tool to qualitatively measure adequate infant and young child feeding practices, and the FCI is also a tool that can measure if children are given opportunities for early learning. Using this Framework, theoretically higher FCI scores would be associated with better IYCF indicators, but this relationship has not yet been assessed.

To assess changes in the dietary practices of children during the period of complementary feeding, the IYCF was measured every 6 months from 6 to 24 months of age. The primary aim of this study was to describe the proportion of children with adequate dietary practices at 6, 12, 18 and 24 months of age and how these practices change over time using the IYCF indicators (i.e., MDD, MMF and MAD) in four LMIC research settings. Our secondary aims were to determine the associations between the IYCF indicators and anthropometric z‐scores from 6 to 24 months, and the association between the IYCF indicators and the FCI at 24 months.

## 2 METHODS

Between 2016 and 2018, all maternal infant dyads in the Women First (WF) Trial, a maternal preconception nutrition supplementation randomized controlled trial, were invited to enrol in the follow‐up growth study at 6 months of age following consent by their mother (Hambidge et al., 2019). In the primary study, mothers were randomised to one of three arms. For Arm 1, participants received a lipid‐based nutrient supplement before conception and throughout pregnancy; Arm 2, the supplement was given during the second and third trimesters only; and Arm 3 did not receive any study supplements, only standard of care for pregnancy in their respective settings. Additional information about the intervention that the mothers completed can be found in the published protocol paper and primary outcomes papers (Hambidge et al., 2014, 2019). It is not anticipated that the nutrition supplement taken during pregnancy would have an effect on the dietary consumption patterns of the mother's offspring during their first 2 years of postnatal life. Participants were from rural/semi‐rural communities in Sud‐Ubangi, Democratic Republic of Congo (DRC); Chimaltenango, Guatemala; Belagavi, North Karnataka, India; and Thatta, Sindh Province, Pakistan. Two anthropometrists from the WF assessment teams visited the mother/child dyad at their home or at the local health centre to obtain anthropometric measurements and assess dietary patterns longitudinally at 6, 12, 18 and 24 months of age (WHO and United Nations Children's Fund UNICEF, 2021). The FCI questionnaire was administered at 24 months of age.

### 2.1 Infant and young child feeding indicators

To calculate the IYCF score, mothers were interviewed using a questionnaire about infant feeding practices during the preceding 24 h of the study visit (WHO, 2010). MDD was calculated as the percentage of children who received food from five or more of eight food groups on the previous day. The eight food groups were: (1) human milk; (2) grains, roots, tubers; (3) nuts and pulses; (4) milk and dairy; (5) meat, fish, insects; (6) eggs; (7) vitamin‐A rich fruits and vegetables and (8) other fruits and vegetables.

MMF was calculated as the percentage of breastfed children who consumed solid, semi‐solid or soft foods at least three times in the previous day. For nonbreastfed children, MMF was calculated as those who received at least four solid, semi‐solid or soft foods or milk feeds during the previous day, and at least one of those meals was either solid, semi‐solid or soft food. MAD was calculated as the proportion of children who consumed at least the MDD and the MMF during the previous day and were either breastfed or consumed the minimum milk feeding frequency (at least two milk feeds/day) (WHO and United Nations Children's Fund UNICEF, 2021). To assess the consumption of ASF and its associations with length‐for‐age z‐score (LAZ), we combined the three food groups milk/dairy, meat/fish/insects and eggs into one group titled ASF group.

### 2.2 Anthropometric indicators

Weight, length and head circumference were obtained by a highly trained assessment team at each site utilising standardised calibrated study equipment at the local health centres or in the participants' homes (Krebs et al., 2021). Anthropometric measurements were converted to age‐ and sex‐ specific z‐scores using the 2006 WHO child growth standards. Z‐scores that were outside of the biologically plausible range were excluded (WHO, 2006).

### 2.3 Family care indicators

The FCI questionnaire (Supporting Information: Table 1) was administered by the assessment team only at the 24 months visit (Hamadani et al., 2010). The answers to the questions were dichotomous, for example, 1 = the presence of books in the household or 0 = absence of books in the household. Questions were categorised into four groups for analysis: (1) play activities (10 questions), (2) variety of play materials (8 questions), (3) sources of play materials (3 questions) and (4) household books (1 question). The sub‐totals were calculated for each of the four groups. The total sum was calculated for all four groups with a maximum possible score of 22 used for this analysis. A greater score is indicative of more family stimulation for and interaction with the child.

### 2.4 Ethics approval

The WF trial was approved by the Colorado Multiple Institutional Review Board; Comité de Ética Universidad Francisco Marroquin; Jawaharlal Nehru Medical College Institutional Ethics Committee on Human Subjects Research and the Indian Council of Medical Research; Comité D'Ethique, Ecole De Sante Publique, University of Kinshasa; the Aga Khan University Ethical Review Committee; and RTI International (North Carolina, USA). Mothers provided written informed consent for themselves and their children.

### 2.5 Statistical analysis

Baseline characteristics and the IYCF indicators at each time point (6, 12, 18 and 24 months) were described using frequency and percentage for nominal variables and measures of central tendency and dispersion for continuous variables. Linear regressions were performed to assess trends of each IYCF indicator and each food group from 6 to 24 months of age for all sites combined and by individual site. Line plots and Equiplots were used to visually display the percentage of children that met the criteria for each IYCF indicator and food group (Equiplot—International Centre for Equity in Health | Pelotas, 2023).

Poisson regressions were performed to evaluate the association between the FCI (total and subscores) and each IYCF indicator at 24 months of age. Multilevel mixed‐effects linear regression models were used to assess associations of anthropometric z‐scores from 6, 12, 18 and 24 months with each IYCF indicator. In the multilevel models for all sites combined, the first level of analysis was the child, the second level was the cluster and the third level was the site. For site analyses, the first level was the child, and the second level was the cluster. Child age was included as an interaction term to assess whether the association of the anthropometric z‐score with each IYCF indicator was modified by child age.

A stepwise backward elimination approach was applied to identify covariates based on a conceptual hierarchical model with three levels (Victora et al., 1997). Covariates with a *p* < 0.05 remained in the final model. In this article, a list of covariates for each model is included in the footnote of each respective table. The intervention arm and sex of the child remained in the final models regardless of the outcome of the stepwise approach. Mothers in the primary maternal nutrition study were randomised to three intervention arms: Arm 1: nutrition supplementation initiated before preconception; Arm 2: nutrition supplementation initiated during early pregnancy; or Arm 3: control. While it was not anticipated that the treatment arm would affect the dietary patterns of the children postnatally, the treatment arm was included in the model to ensure that it had no effect on this study's outcomes. Final models were adjusted for multiple comparisons using Bonferroni's method and reported as contrasts (differences) of margins along with significance tests and confidence intervals for the contrasts. A *p* for an interaction <0.2 was considered significant. For all the other analyses, a ≤0.05 was considered statistically significant. To measure a significant change in the percent frequency of the IYCF indicators over time, a *p* trend ≤0.05 was considered significant (Patino & Ferreira, 2016). All analyses were performed using Stata 16.1 (StataCorp).

## 3 RESULTS

**Figure 1:**
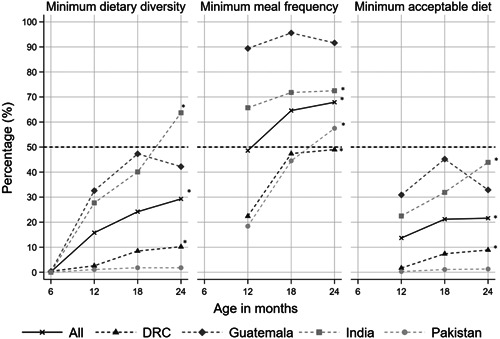
Percentage of adequate infant and young child feeding (IYCF) practices at 6, 12, 18 and 24 months and trends over time by all sites and each site. Minimum dietary diversity, minimum meal frequency and minimum acceptable diet during the previous day were calculated following the updated definition published by the WHO/UNICEF Technical expert advisory group on nutrition monitoring (TEAM) in 2021 (WHO and United Nations Children's Fund UNICEF, 2021). The trend of each IYCF indicator from 12 to 24 months of age for all sites combined and individual sites was assessed using linear regression models. A significant linear trend (trend *p* < 0.05) in the percentages of consumption from 6 to 24 months for MDD and 12−24 for MMF and MAD of a specific IYCF indicator was denoted with an asterisk (*). DRC, Democratic Republic of Congo; WHO, World Health Organisation.

A total of 2413 mother and child dyads: 570 from DRC, 614 from Guatemala, 589 from India and 640 from Pakistan were included in the analysis. Descriptive analysis of demographic and SES characteristics of the dyads and the household are presented in Supporting Information: Table 2. There was an even split of males and females enroled across the sites. The average age of mothers was 23.2 ± 4.2 years, and the average BMI was 21.5 ± 4.0 for all sites combined. The frequency of adequate MDD, MMF and MAD increased with age when all sites were combined, but the overall percentage of children who met the MAD requirements at 24 months remained below 50% (Figure 1). When stratified by site, the number of children who met the MDD, MMF and MAD in the DRC and India significantly increased over time. In Pakistan, only the MMF significantly increased from 12 to 24 months, 18% to 57%, respectively, (*p* trend <0.001), and in Guatemala, only the MDD significantly increased from nearly 0% at 6 months to 42% at 24 months, (*p* trend = 0.001).

**Figure 2:**
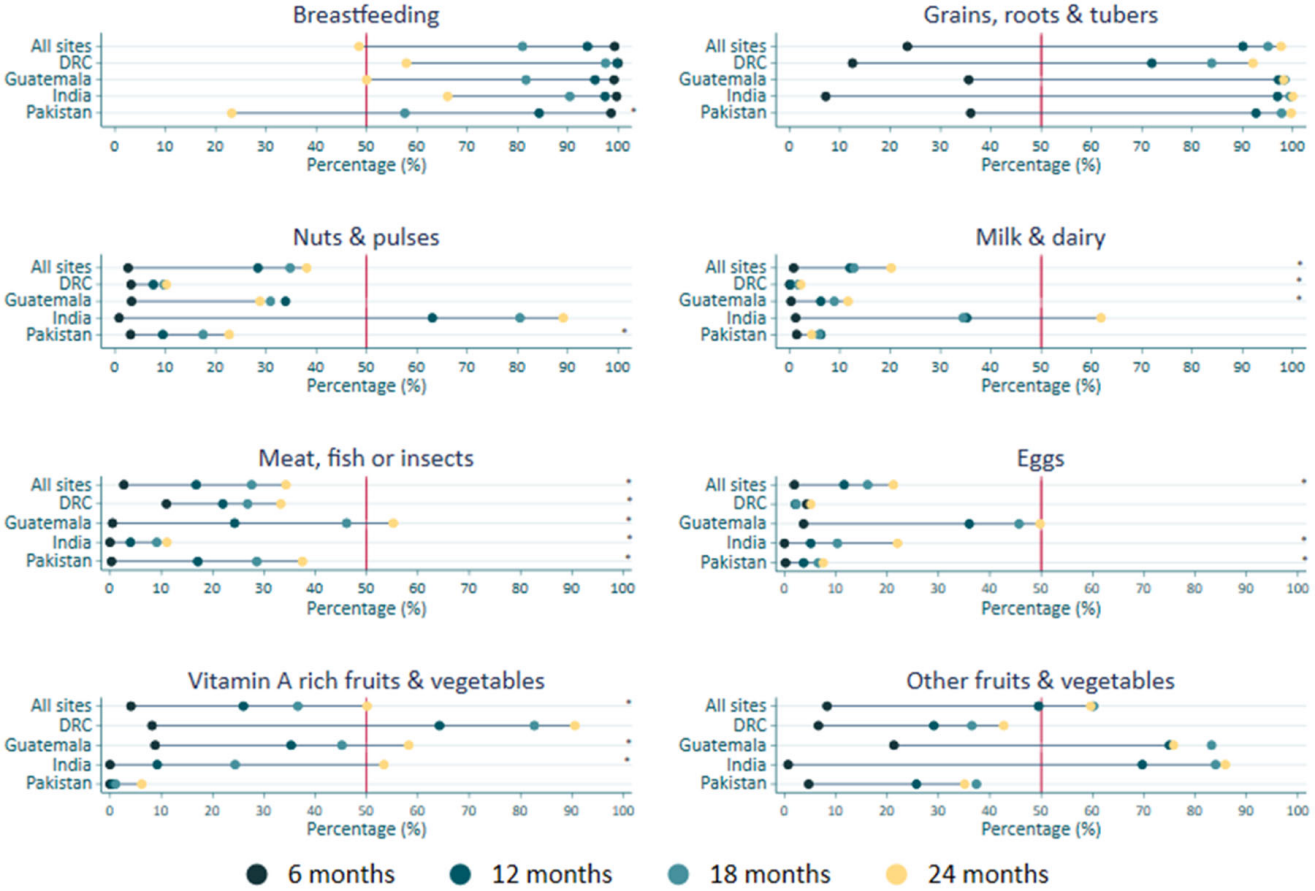
Percentages and trends of food groups consumption from 6 to 24 months of age by all sites and each site. The percentage of consumption of food groups from 6 to 24 months of age of each food group for all sites combined and individual sites was assessed using linear regression models. A significant linear trend (trend *p* < 0.05) in the percentages of consumption from 6 to 24 months of a specific infant young child feeding indicator was denoted with an asterisk (*) at the end of the line that corresponds to the country. DRC, Democratic Republic of Congo.

Analysis of the diet by food groups revealed that 92−100% of children consumed grains, roots and tubers during the previous day at 24 months of age (Figure 2). Consumption of the meat, fish and insect food group significantly increased from 6 to 24 months of age (*p* trend <0.001), but the overall frequency remained low at 24 months ranging from 11% to 55% across the sites. Consumption of vitamin A‐rich fruits and vegetables followed a similar trend, ranging from 6% to 58%, except for the DRC, which had a 90% frequency of consumption at 24 months. Breastfeeding decreased across all sites from 6 to 24 months.

Examination of the IYCF indicators and z‐scores (LAZ, weight‐for‐age z‐score, head circumference‐for‐age z‐score) at 6, 12, 18 and 24 months did not show any significant correlations except for MDD and MMF with LAZ at 24 months (Table 1). For all sites combined, MDD (*β* = 0.12, 95% CI 0.04−0.22) and MMF (*β* = 0.10, 95% CI 0.03−0.17) were positively associated with LAZ at 24 months. By individual site, MMF of children from the Guatemala site at 24 months was positively associated with LAZ (*β* = 0.22, 95% CI 0.04−0.40). ASF were positively associated with LAZ at 24 months for all sites combined (*β* = 0.10, 95% CI 0.03−0.18) (Table 2).

**Table 1:** Association between length‐for‐age z‐scores and infant and young children indicators of children at 6, 12, 18 and 24 months.

	Combined sites^a^		Democratic Republic of the Congo^b^		Guatemala^b^		India^b^		Pakistan^b^	
Child age	*n*	*β* (95% CI)	*n*	*β* (95% CI)	*n*	*β* (95% CI)	*n*	*β* (95% CI)	*n*	*β* (95% CI)
*Minimum dietary diversity*										
6 months	2296	−0.08 (−0.79 to 0.64)	539	−0.02 (−1.42 to 1.38)	566	0.05 (−0.80 to 0.90)	573		618	−1.26 (−3.05 to 0.53)
12 months	2296	0.00 (−0.10 to 0.10)	539	−0.20 (−0.73 to 0.34)	566	−0.06 (−0.19 to 0.08)	573	0.03 (−0.11 to 0.18)	618	0.00 (−0.68 to 0.68)
18 months	2296	0.05 (−0.04 to 0.14)	539	0.10 (−0.21 to 0.41)	566	0.00 (−0.12 to 0.13)	573	−0.04 (−0.18 to 0.09)	618	0.22 (−0.32 to 0.77)
24 months	2296	0.12 (0.04 to 0.22)*	539	0.19 (−0.10 to 0.48)	566	0.07 (−0.05 to 0.20)	573	−0.10 (−0.24 to 0.04)	618	0.14 (−0.40 to 0.68)
*Minimum meal frequency*										
12 months	2296	−0.05 (−0.12 to 0.01)	539	−0.08 (−0.26 to 0.11)	566	−0.02 (−0.18 to 0.14)	573	0.04 (−0.09 to 0.18)	618	−0.02 (−0.17 to 0.13)
18 months	2296	0.01 (−0.06 to 0.08)	539	−0.04 (−0.20 to 0.11)	566	0.03 (−0.22; 0.27)	573	0.04 (−0.10; 0.18)	618	0.07 (−0.05; 0.19)
24 months	2296	0.10 (0.03 to 0.17)*	539	0.01 (−0.14 to 0.17)	566	0.22 (0.04 to 0.40)*	573	0.04 (−0.11 to 0.18)	618	0.08 (−0.03 to 0.20)
*Minimum acceptable diet*										
12 months	2296	−0.07 (−0.16 to 0.02)	539	−0.33 (−0.92 to 0.25)	566	−0.08 (−0.19 to 0.03)	573	0.04 (−0.09 to 0.17)	618	0.88 (−0.13 to 1.90)
18 months	2296	−0.00 (−0.08 to 0.07)	539	0.04 (−0.26 to 0.33)	566	−0.01 (−0.11 to 0.09)	573	0.01 (−0.11 to 0.13)	618	0.09 (−0.46 to 0.63)
24 months	2296	0.06 (−0.02 to 0.14)	539	0.07 (−0.21 to 0.34)	566	0.07 (−0.04 to 0.18)	573	−0.00 (−0.12 to 0.11)	618	0.07 (−0.44 to 0.58)

*Note*: A significant *β* was denoted by an asterisk (*) in the 95% CI (*p* < 0.05). The *n* in this table accounts for about <10% loss to follow‐up over the longitudinal period. Multilevel mixed‐affects linear regression models were applied to assess the association of the infant young child feeding indicators (IYCF) and length‐for‐age z‐score (LAZ) at 6, 12, 18 and 24 months of age by all sites and individual sites.

Abbreviations: 95% CI, 95% confidence interval; *β*, Coefficient of the multilevel mixed‐effects linear regression model.

^a^ The first level of analysis was the child, the second level was the cluster and the third level was the site.

^b^ Two levels were considered for analysis: the child (first level) and the cluster (second level). All models were adjusted by socioeconomic status tally indicators, treatment arm, maternal education at enrolment, maternal height and body mass index at enrolment, mode of delivery, sex of the child and LAZ at 6 months. The p‐value for the interaction of a specific IYCF indicator and child age was <0.02. LAZ was calculated using the WHO Child Growth Standards (WHO, 2006). Minimum dietary diversity, minimum meal frequency and minimum acceptable diet during the previous day were calculated following the updated definition published by the WHO/UNICEF Technical expert advisory group on nutrition monitoring (TEAM) in 2021 (WHO and United Nations Children's Fund UNICEF, 2021). Note: Minimum meal frequency and minimum adequate diet were not measured at 6 months because at 6 months the 2013 version of the IYCF tool was used.

**Table 2:** Associations between length‐for‐age z‐scores and animal source food groups consumption of children at 6, 12, 18 and 24 months.

	Combined sites^a^		Democratic Republic of the Congo^b^		Guatemala^b^		India^b^		Pakistan^b^	
Child age	*n*	*β* (95% CI)	*n*	*β* (95% CI)	*n*	*β* (95% CI)	*n*	*β* (95% CI)	*n*	*β* (95% CI)
6 months	2296	0.07 (−0.10 to 0.23)	526	−0.12 (−0.36 to 0.13)	566	0.23 (−0.07 to 0.53)	573	0.07 (−0.50 to 0.64)	618	−0.06 (−0.61 to 0.50)
12 months	2296	−0.02 (−0.10 to 0.06)	526	−0.02 (−0.22 to 0.18)	566	0.00 (−0.12 to 0.12)	573	0.02 (−0.11 to 0.16)	618	−0.03 (−0.20 to 0.13)
18 months	2296	−0.03 (−0.10 to 0.04)	526	0.06 (−0.13 to 0.25)	566	0.00 (−0.15 to 0.14)	573	−0.06 (−0.20 to 0.07)	618	−0.07 (−0.22 to 0.08)
24 months	2296	0.10 (0.03 to 0.18)*	526	0.08 (−0.11 to 0.26)	566	0.09 (−0.07 to 0.26)	573	0.00 (−0.15 to 0.16)	618	0.03 (−0.12 to 0.18)

*Note*: A significant β was denoted by an asterisk (*) in the 95% CI (*p* < 0.05). Multilevel mixed‐affects linear regression models were applied to assess the association between and animal source food groups consumption and length‐for‐age z‐score (LAZ) at 6, 12, 18 and 24 months of age by all sites and individual sites.

Abbreviations: 95% CI, 95% confidence interval; *β*, Coefficient of the multilevel mixed‐effects linear regression model.

^a^ The first level of analysis was the child, the second level was the cluster and the third level was the site.

^b^ Two levels were considered for analysis: the child (first level) and the cluster (second level). Models were adjusted by socioeconomic status tally indicators, treatment arm, maternal education at enrolment, maternal height, maternal body mass index at enrolment, mode of delivery, sex of the child and LAZ at 6 months. LAZ was calculated using the World Health Organisation (WHO) Child Growth Standards (WHO, 2006). Animal source foods were defined as the proportion of children who received milk/dairy, meat/fish/insects or eggs.

The mean total FCI score ranged from 11.1 to 15.2, out of a total of 22 points across the four sites with Guatemala having the highest mean total FCI (15.2 ± 3.1) and Pakistan the lowest (11.1 ± 3.2) (Supporting Information: Table 3). When the predictive relationship between the mean total FCI and each IYCF indicator was assessed for all sites combined, a significant positive association was found for each of the three IYCF indicators and the mean total FCI: MDD (proportion ratio [PR] = 1.04, 95% CI 1.02−1.07); MMF (PR = 1.02, 95% CI 1.01−1.04); MAD (PR = 1.05, 95% CI 1.02−1.08) (Table 3).

**Table 3:** Associations between the family care indicators and infant and young children feeding (IYCF) indicators of children at 24 months.

	Combined sites		Democratic Republic of the Congo		Guatemala		India		Pakistan	
IYCF indicator	*N*	PR (95% CI)	*N*	PR (95% CI)	*N*	PR (95% CI)	*N*	PR (95% CI)	*N*	PR (95% CI)
*Family care indicator total score*										
Minimum dietary diversity^a^	2190	1.04 (1.02−1.07)*	489	1.10 (1.02−1.18)*	550	1.08 (1.03−1.13)*	556	1.03 (0.99−1.08)	595	1.21 (0.97−1.52)
Minimum meal frequency^b^	2193	1.02 (1.01−1.04)*	489	1.00 (0.97−1.04)	551	1.01 (0.98−1.04)	556	0.99 (0.95−1.03)	597	1.06 (1.02−1.09)*
Minimum acceptable diet^a^	2190	1.05 (1.02−1.08)*	489	1.08 (1.00−1.17)*	550	1.09 (1.03−1.15)*	556	1.02 (0.98−1.08)	595	1.19 (0.92−1.55)
*Play activities sub score*										
Minimum dietary diversity^a^	2190	1.04 (1.00−1.08)	489	1.24 (1.07−1.45)*	550	1.12 (1.04−1.21)*	556	1.04 (0.98−1.11)	595	1.30 (0.95−1.80)
Minimum meal frequency^b^	2193	1.03 (1.00−1.05)	489	1.02 (0.95−1.09)	551	1.01 (0.97−1.06)	556	1.00 (0.94−1.06)	597	1.08 (1.02−1.14)*
Minimum acceptable diet^a^	2190	1.07 (1.02−1.12)*	489	1.24 (1.05−1.46)*	550	1.14 (1.05−1.24)*	556	1.04 (0.97−1.12)	595	1.33 (0.91−1.94)
*Variety of play material sub score*										
Minimum dietary diversity^a^	2190	1.12 (1.06−1.18)*	489	1.11 (0.95−1.30)	550	1.14 (1.03−1.26)*	556	1.05 (0.95−1.15)	595	1.24 (0.73−2.13)
Minimum meal frequency^b^	2193	1.04 (1.01−1.08)*	489	0.99 (0.92−1.07)	551	1.01 (0.95−1.08)	556	0.94 (0.86−1.02)	597	1.11 (1.02−1.21)*
Minimum acceptable diet^a^	2190	1.10 (1.03−1.17)*	489	1.06 (0.89−1.26)	550	1.14 (1.02−1.27)*	556	0.97 (0.87−1.09)	595	1.23 (0.66−2.29)
*Sources of play materials sub score*										
Minimum dietary diversity^a^	2190	1.08 (0.95−1.22)	489	1.32 (0.93−1.86)	550	1.21 (0.90−1.61)	556	1.04 (0.86−1.27)	595	1.99 (0.69−5.73)
Minimum meal frequency^b^	2193	1.07 (0.99−1.16)	489	1.04 (0.90−1.20)	551	0.95 (0.79; 1.13)	556	1.04 (0.87−1.25)	597	1.11 (0.96−1.30)
Minimum acceptable diet^a^	2190	1.10 (0.95−1.28)	489	1.29 (0.89−1.87)	550	1.12 (0.82−1.54)	556	1.08 (0.85−1.37)	595	1.32 (0.46−3.80)
*Household books sub score*										
Minimum dietary diversity^a^	2190	1.02 (0.95−1.10)	489	1.15 (0.97−1.36)	550	1.06 (0.95−1.18)	556	1.07 (0.89−1.28)	595	‐c
Minimum meal frequency^b^	2193	1.05 (1.00−1.11)	489	1.06 (0.96−1.17)	551	1.01 (0.93−1.10)	556	1.07 (0.92−1.25)	597	1.06 (0.72−1.56)
Minimum acceptable diet^a^	2190	1.07 (1.00−1.16)	489	1.15 (0.96−1.38)	550	1.10 (0.98−1.23)	556	1.15 (0.96−1.39)	595	‐c

*Note*: A significant PR was denoted by an asterisk (*) in the 95% confidence interval (*p* < 0.05). Poisson regression was applied to assess the association between family care indicators at 24 months of age and minimum dietary diversity, minimum meal frequency or minimum acceptable diet by all sites and individual sites (WHO and United Nations Children's Fund UNICEF, 2021). The prevalence rate ratio estimation option was applied to report eβi rather than βi. The standard errors and confidence intervals were similarly transformed. In the model, the infant young child feeding indicator was a binary outcome while the family care indicator was a discrete explanatory variable.

Abbreviations: 95% CI, 95% confidence interval; PR, proportion ratio.

^a^ Model adjusted by socioeconomic status tally indicators, treatment arm, maternal education at enrolment, interval from last pregnancy, mode of delivery and sex of the child.

^b^ Model adjusted by socioeconomic status tally indicators, treatment arm, maternal education at enrolment, maternal body mass index at enrolment and sex of the child.

^c^ Result is not shown because the model did not converge due to a lack of sample size and the low prevalence of the IYCF indicators.

## 4 DISCUSSION

The most salient finding from this analysis was that while across all sites combined, the proportion of children meeting the three IYCF indicators increased as they aged, the number of children meeting MAD remained below 40% at 24 months of age. We also found that as the proportion of children meeting MDD and MFF improved, there was a positive association with LAZ. Additionally, the total FCI score was positively associated with all three IYCF indicators.

The persistently low MAD was driven primarily by the smaller proportion of children meeting the MDD throughout this study. Only in India did MDD exceed 50% by 24 months; at all the other sites the MDD ranged from 2% to 42%. The greatest increase in MDD and MMF occurred from 12 to 18 months of age, and by 24 months, three out of the four sites had over 50% of the children meeting MMF; only DRC was below 50%. Our findings mirror those of a larger 80 country survey completed in LMICs that found that less than 25% of countries surveyed had MDD and MAD prevalence above 50% (Gatica‐Dominguez et al., 2021). Our study sites were low‐resource settings, and prior work suggests that overall economic improvement may be an important aspect in making impactful improvements to children's diets (Baye & Kennedy, 2020; Gatica‐Domínguez et al., 2021).

A closer analysis of the dietary diversity at each site showed that grains, roots and tubers were the most consumed food group and comprised most of the diet. In contrast, consumption of additional food groups that are nutrient‐rich and add diversity, such as meat, fish or insects; vitamin A‐rich fruits and vegetables; eggs; nuts and pulses; and milk and dairy, varied among the sites and were less frequently eaten compared to staple foods (Figure 2). The children in the Pakistan site had the lowest dietary diversity and by 24 months only grains, roots and tubers exceeded 50% with very low intakes (<10%) of milk and dairy; eggs; and vitamin A‐rich fruits and vegetables. While children at the DRC site had low intakes of many of the nutrient‐dense food groups, they had very high intakes (>50%) of vitamin A‐rich fruits and vegetables. By 24 months, the consumption was over 90%, consistent with the community's heavy reliance on cassava leaves and other dark leafy greens and recipes with red palm oil, all of which contribute to vitamin‐A intake (Lander et al., 2019). The findings that India had the greatest increase in the number of food groups eaten from 6 to 24 months but flesh foods and eggs remained below 50%, and that Guatemala had the highest intakes of flesh foods and eggs by 24 months of age support previous national survey findings (Ricardo et al., 2023).

This study found a positive correlation between MDD and LAZ at 24 months and between MMF and LAZ at 24 months. Observational studies have found that an inadequate child MDD is associated with stunting (Anin et al., 2020; Perkins et al., 2018; Rakotomanana et al., 2017; Wang et al., 2017). While it is well established that stunting is a multifactorial problem, early consumption of ASF that provide high‐quality proteins and rich sources of important micronutrients have been positively associated with growth (Krebs et al., 2011). By 24 months of age, the average consumption of ASF ranged from 20% to 34% for all sites combined. Our findings matched previous research reporting that consumption of ASF, especially flesh foods and eggs, were low globally for children of complementary feeding age (Krebs et al., 2011; Ricardo et al., 2023). When we combined the ASF groups (meat, fish, insects; eggs; milk and dairy) and examined the relationship with LAZ, we observed a positive association at 24 months (Table 2). This suggests that an emphasis on consuming ASF may be an important part of diets to promote healthy growth and development. This contrasts with the findings from a 2019 Cochrane review of intervention trials that did not find an association between intake of ASF and growth (Eaton et al., 2019). The data from the Cochrane review were quite heterogenous and rated as low quality overall. Findings from high‐resource settings regarding the impact of ASF on growth are also mixed (English et al., 2019). Overall, these varied findings about consumption of ASF on growth warrant additional research, especially in settings with high rates of growth faltering and other vulnerabilities.

To our knowledge, this is the first time the IYCF indicators have been assessed in relation to FCI. The mean total FCI score and, separately, the variety of play materials were positively associated with each of the three IYCF indicators at 24 months of age for all sites combined. India was the only site with no associations between any of the FCI and IYCF indicators. While previous studies have not looked at FCI in relation to nutrition, published data have reported positive associations between FCI scores and IQ and other indicators of early child development (Rubio‐Codina & Grantham‐McGregor, 2020; Zhong & Luo, 2020). Similarly, previous research on responsive feeding found associations with better child nutrition (Black & Aboud, 2011). The FCI measures how much stimulation a child receives from family interactions. Therefore, it may be appropriate to consider the FCI as a proxy for nurturing care. In our study, children whose families reported more opportunities to interact with their children also had greater intake of diverse foods.

The FCI was only assessed cross‐sectionally at 24 months, so this analysis was not able to identify if the FCI or IYCF indicators had a greater impact on linear growth. It is possible that it may not be one or the other that influences linear growth but rather a synergy of both. This is highlighted in the Nurturing Care Framework that states adequate nutrition, responsive caregiving and opportunities for learning, good health and safety and security are needed for optimal child development (United Nations Children's Fund UNICEF and WHO, 2022).

Strengths of this study include its longitudinal design and conduct in four disparate study locations with similar results found across the sites, making the results from this study generalisable to young children living in LMIC. While the IYCF indicators are usually applied to national representative health and nutrition surveys, as well as larger epidemiological datasets (e.g., cohort studies), our findings suggest that using IYCF indicators in smaller intervention trials is appropriate because it was sensitive enough to identify differences across sites and changes within the site even with a smaller sample size. Another strength of this study is that the dietary data were collected by highly trained staff under the supervision of experienced nutritionists.

This study was not without limitations. The IYCF indicators qualitatively assess the child's dietary patterns in terms of diversity and frequency of consumption of predetermined food groups, so the qualitative nature of the IYCF tool precluded the estimation of individual nutrient intakes. Also, the IYCF indicators are calculated from the foods a child ate during the previous 24‐h period, so it is impossible to know if the data collected in the past 24‐h were representative of usual intake. However, the generally limited resources in the study sites resulted in relatively monotonous diets, enhancing the likelihood that our findings are representative of typical intakes (Krasevec et al., 2017).

In conclusion, this study demonstrates that children from diverse communities in DRC, Guatemala, India and Pakistan had poor complementary feeding practices from 6 to 24 months of age. The homogeneity of the diets, which included the low intakes of ASF groups during the 6–12‐month period, highlights an opportunity for improving children's diets. Increasing intake of a variety of fruits, vegetables and legumes would further improve overall diet quality. In communities where consumption of ASF is common for older children and adults, the inclusion of meats, fish and eggs offers a nutrient‐rich opportunity for this younger age group to improve diet quality, and possibly overall growth, as suggested by the positive association between ASF and meal frequency consumption and linear growth status at 24 months. In addition, the positive association between the IYCF indicators and the FCI suggests that the nurturing care framework that includes attentive caregiving and adequate diets is important to promote optimal child nutrition and growth. In total, our findings provide evidence that the FCIs represent important caregiver behaviours and child interactions that are, in turn, associated with higher‐quality infant and young child feeding.

## AUTHOR CONTRIBUTIONS

Nancy F. Krebs and K. Michael Hambidge designed the research study. Jamie E. Westcott, Rebecca Lander, Antoinette K. Tshefu, Sangappa M. Dhaded, Sunil S. Vernekar, Veena R. Herekar, Sarah Saleem, Elizabeth M. McClure, Abhik Das, Vanessa R. Thorsten, Richard J. Derman, Robert L. Goldenberg, Carl Bose, Melissa S. Bauserman and Marion W. Koso‐Thomas had scientific input into the study design and interpretation of results. Julie M. Long, Gabriela Tejeda, Tshilenge S. Diba, Shivanand C. Mastiholi, Umber S. Khan, Ana Garcés, Lester Figueroa, Adrien Lokangaka, Shivaprasad S. Goudar and Sumera Aziz Ali performed the research. Giovanna Gatica‐Domínguez analysed the data. Julie M. Long, Giovanna Gatica‐Domínguez, Douglas Taren, Nancy F. Krebs and K. Michael Hambidge wrote the paper. All authors have read and approved the final manuscript.

## WOMEN FIRST PRECONCEPTION MATERNAL NUTRITION TRIAL GROUP

Rebecca Lander (rebecca.lander3@gmail.com); Antoinette K. Tshefu (Kinshasa School of Public Health, Kinshasa, DRC); Sangappa M. Dhaded, Sunil S. Vernekar and Veena R. Herekar (Women's and Children's Health Research Unit, KLE Academy of Higher Education and Research's Jawaharlal Nehru Medical College, Belagavi, India); Sarah Saleem (Department of Community Health Sciences, Aga Khan University, Karachi, Pakistan); Elizabeth M. McClure, Abhik Das and Vanessa R. Thorsten (RTI International, Durham, NC, USA); Richard J. Derman (Thomas Jefferson University, Philadelphia, PA, USA); Robert L. Goldenberg (Columbia University, New York, NY, USA); Carl Bose and Melissa S. Bauserman (University of North Carolina at Chapel Hill, Chapel Hill, NC, USA); Marion W. Koso‐Thomas (Pregnancy and Perinatology Branch, NICHD/NIH, Bethesda, MD, USA).

## CONFLICT OF INTEREST STATEMENT

The authors declare no conflict of interest.

## Supporting information

Supporting information.

## Data Availability

The data that support the findings of this study are available from the corresponding author upon reasonable request.
