# Supplementary material for: Infant and young child feeding indicators are positively associated with length and family care indicators in the children of the Women First trial participants
Source: Matern Child Nutr. 2023 Oct 10;20(1):e13572. doi: 10.1111/mcn.13572 (PMC10750017; doi:10.1111/mcn.13572)
Supplement: Supplementary file 1 — Supporting information. [file MCN-20-e13572-s001.docx]

**Supplementary Table 1.** Family care indicators survey questions

| **Reading Materials in the Home** | | |
| --- | --- | --- |
| 1. Do you have children’s books or picture books at home for your child? | Yes  No  Don’t know/refuse | |
| 1. How many books are there? | ______________ | |
| **Play Items in the Home**  I am interested in learning about the thigs that your child plays with when he/she is at home: | | |
| 1. Does he/she play with home-made toys? (e.g. balls made of plastic papers, clay dolls, wire cars, or other toys made at home) | Yes  No  Don’t know/refuse | |
| 1. Does he/she play with toys from a shop or manufactured toys? (e.g. balls, dolls, cars, or other toys from a shop) | Yes  No  Don’t know/refuse | |
| 1. Does he/she play with household objects (such as bowls, spoons, matchboxes, empty bottles or pots) or objects found outside? (e.g. as sticks, rocks, animal shells or leaves) | Yes  No  Don’t know/refuse | |
| 1. Does he/she play with things that make or play music? (e.g. toy phone, toy radio, doll that plays music) | Yes  No  Don’t know/refuse | |
| 1. Does he/she play with things for drawing or writing? (e.g. pencils, pens, charcoal) | Yes  No  Don’t know/refuse | |
| 1. Does he/she play with picture books for children (not school books)? | Yes  No  Don’t know/refuse | |
| 1. Does he/she play with things meant for stacking, constructing, or building? (e.g. blocks, maize cobs to build a pretend house) | Yes  No  Don’t know/refuse | |
| 1. Does he/she play with things for moving around? (e.g. balls, cars with a handle, toy bicycles, kites) | Yes  No  Don’t know/refuse | |
| 1. Does he/she play with toys for learning shapes (circle, square, and triangle) and colors? | Yes  No  Don’t know/refuse | |
| 1. Does he/she play with things for pretending? (e.g. dolls, tins to pretend to cook) | Yes  No  Don’t know/refuse | |
| 1. Does he/she use ‘apps’ on a phone, tablet, or computer or watch videos or TV programs that are specific for helping the child learn new things? | Yes  No  Don’t know/refuse | |
| **Interaction in the Home**  In the past 3 days, did you or any household member over 9 years of age engage with any of the following activities with your child. NOTE: An adult is anyone 15 years of age or older. | | |
| 1. In the past 3 days, has anyone in your household read books to or looked at picture books with your child? | Yes  No  Don’t know/refuse | Mother  Father  Other adult  Child ≥ 9 yrs |
| 1. In the past 3 days, had anyone in your household told stories to your child? | Yes  No  Don’t know/refuse | Mother  Father  Other adult  Child ≥ 9 yrs |
| 1. In the past 3 days, has anyone in your household sang songs to your child/with your child including lullabies? | Yes  No  Don’t know/refuse | Mother  Father  Other adult  Child ≥ 9 yrs |
| 1. In the past 3 days, has anyone in your household taken your child outside the home compound or yard or enclosure? “visiting’ no e.g. to the hospital. | Yes  No  Don’t know/refuse | Mother  Father  Other adult  Child ≥ 9 yrs |
| 1. In the past 3 days, has anyone in your household played with your child? | Yes  No  Don’t know/refuse | Mother  Father  Other adult  Child ≥ 9 yrs |
| 1. In the past 3 days, has anyone in your household counted or drawn things to or with your child? | Yes  No  Don’t know/refuse | Mother  Father  Other adult  Child ≥ 9 yrs |
| 1. In the past 3 days, has anyone in your household helped your child learn letters? (e.g. the alphabet song) | Yes  No  Don’t know/refuse | Mother  Father  Other adult  Child ≥ 9 yrs |
| 1. In the past 3 days, has anyone in your household constructed objects from paper, wire or mud with your child? (e.g. made paper airplanes, small toys or objects with child) | Yes  No  Don’t know/refuse | Mother  Father  Other adult  Child ≥ 9 yrs |
| 1. In the past 3 days, has anyone in your household taught your child the names of people or objects, or use of objects? (e.g. family members, food, objects, dishes or utensils, clothing, colors, shapes, toys, or body parts, or teaches child things like matches are for lighting fire, brooms are for sweeping, etc.)? | Yes  No  Don’t know/refuse | Mother  Father  Other adult  Child ≥ 9 yrs |
| 1. In the past 3 days, has anyone in your household taught your child names of things OUTSIDE of your home (e.g. animals, plants, rain, sun, mud insects, moon, sky, bodies, of water, roads, cars, bicycles, etc.) | Yes  No  Don’t know/refuse | Mother  Father  Other adult  Child ≥ 9 yrs |

**Supplementary Table 2.** Maternal and child characteristics by all sites combined and each site individually from the Women First Preconception Trial

| **Characteristics** | **Combined sites**  **N = 2413**  **n (%)** | **Democratic Republic of the Congo**  **N = 570**  **n (%)** | **Guatemala**  **N = 614**  **n (%)** | **India**  **N = 589**  **n (%)** | **Pakistan**  **N = 640**  **n (%)** |
| --- | --- | --- | --- | --- | --- |
| *Child* |  |  |  |  |  |
| Sex  Female  Male | 1218 (50.5)  1195 (49.5) | 281 (49.3)  289 (50.7) | 307 (50.0)  307 (50.0) | 293 (49.8)  296 (50.3) | 337 (52.7)  303 (47.3) |
| *Maternal* |  |  |  |  |  |
| Age, years (mean ± SD)  <20  20-24  25+ | 23.2 ± 4.2  496 (20.6)  1002 (41.5)  915 (37.9) | 22.9 ± 4.6  143 (25.1)  222 (38.9)  205 (36.0) | 24.3 ± 4.4  94 (15.3)  243 (39.6)  277 (45.1) | 22.0 ± 3.4  146 (24.8)  318 (54.0)  125 (21.2) | 23.8 ± 4.1  113 (17.7)  219 (34.2)  308 (48.1) |
| Parity  0 (Nulliparous)  1  2+ | 488 (20.2)  761 (31.5)  1164 (48.3) | 115 (20.2)  136 (23.9)  319 (55.9) | 39 (6.4)  233 (37.9)  342 (55.7) | 151 (25.6)  244 (41.5)  194 (32.9) | 183 (28.6)  148 (23.1)  309 (48.3) |
| Education attained  No formal schooling  Primary  Secondary or more | 763 (31.6)  914 (37.9)  736 (30.5) | 130 (22.8)  345 (60.5)  95 (16.7) | 44 (7.2)  414 (67.4)  156 (25.4) | 46 (7.8)  92 (15.6)  451 (76.6) | 543 (84.8)  63 (9.9)  34 (5.3) |
| BMI, kg/m^2^ (mean ± SD)  <18.5  18.5-24.9  25.0< | 21.5 ± 4.0  555 (23.0)  1454 (60.3)  403 (16.7) | 20.6 ± 2.6  99 (17.4)  444 (77.9)  27 (4.7) | 25.4 ± 4.1  8 (1.3)  311 (50.7)  294 (48.0) | 20.0 ± 3.4  221 (37.5)  319 (54.2)  49 (8.3) | 19.7 ± 2.9  227 (35.5)  380 (59.3)  33 (5.2) |
| Minimum diet diversity^‌^^†^  Inadequate  Adequate | 527 (67.2)  257 (32.8) | 137 (83.0)  28 (17.0) | 121 (62.7)  72 (37.3) | 73 (36.1)  129 (63.9) | 196 (87.5)  28 (12.5) |
| Interval between last pregnancy  Never pregnant  12-24 months  ≥ 24 months | 488 (20.3)  602 (25.0)  1320 (54.7) | 115 (20.2)  53 (9.3)  402 (70.5) | 39 (6.4)  94 (15.3)  480 (78.3) | 151 (25.6)  101 (17.2)  337 (57.2) | 183 (28.7)  354 (55.5)  101 (15.8) |
| *Household* |  |  |  |  |  |
| SES at maternal enrollment^††^  None  1-2  3-4  5-6 | 322 (13.3)  681 (28.2)  976 (40.5)  434 (18.0) | 305 (53.5)  256 (44.9)  9 (1.6)  0 (0) | 0 (0)  71 (11.6)  363 (59.1)  180 (29.3) | 0 (0)  59 (10.0)  376 (63.8)  154 (26.2) | 17 (2.7)  295 (46.1)  228 (35.6)  100 (15.6) |

**Note:** SD = standard deviation; BMI = body mass index at enrollment; SES = socioeconomic status

^†^A randomized subset of women from the Women First Trial were selected for dietary assessment at 12 weeks gestation (All sites: N = 784; Democratic Republic of Congo: N = 165; Guatemala: N = 193; India: N = 202; Pakistan: N = 224). Foods and beverages consumed were grouped in ten food categories: 1) Grains, white roots and tubers, and plantains; 2) Pulses (beans, peas and lentils); 3) Nuts and seeds; 4) Dairy; 5) Meat, poultry and fish; 6) Eggs; 7) Dark green leafy vegetables; 8) Other vitamin A-rich fruits and vegetables; 9) Other vegetables; and 10) Other fruits according to the Food and Agriculture Organization of the United Nations guidelines to obtain the maternal minimum diet diversity (MDD-W). MDD-W defined as the proportion of women who have consumed at least five out of ten defined food groups the previous day (Food and Agricultural Organization of the United Nations, 2016). This variable is the average of two 24-hour assessments of women enrolled in the Women First Trial in their first trimester of pregnancy (Lander et al., 2019).

^††^The SES tally provides the number of indicators available from the following list: electricity, improved water source, sanitation, man- made flooring, improved cooking fuels, and household assets.

**Supplementary Table 3.** Family care indicators total score by all sites combines and each site

| **Family care indicator** | **All sites**  **N = 2198** | **Democratic Republic of the Congo**  **N = 494** | **Guatemala**  **N = 551** | **India**  **N = 556** | **Pakistan**  **N = 597** |
| --- | --- | --- | --- | --- | --- |
| Total family care indicators | 12.5 ± 3.7 | 11.5 ± 4.2 | 15.2 ± 3.1 | 12.1 ± 2.7 | 11.1 ± 3.2 |
| Play activities | 5.5 ± 2.0 | 6.1 ± 1.9 | 6.2 ± 1.9 | 5.1 ± 1.8 | 4.8 ± 2.0 |
| Variety of play material | 4.3 ± 1.7 | 3.1 ± 1.9 | 5.7 ± 1.4 | 4.6 ± 1.2 | 3.9 ± 1.4 |
| Sources of play materials | 2.4 ± 0.7 | 2.2 ± 0.9 | 2.8 ± 0.5 | 2.3 ± 0.5 | 2.4 ± 0.7 |
| Household books, n (%)  0  1  2+ | 1771 (80.6)  301 (13.7)  126 (5.7) | 440 (89.1)  19 (3.9)  35 (7.1) | 237 (43.0)  243 (44.1)  71 (12.0) | 504 (90.7)  35 (6.3)  17 (3.1) | 590 (98.8)  4 (0.7)  3 (0.5) |

Values are mean ± SD unless otherwise noted; The validated questionnaire about the family care indicators was administered by the assessment team at the 24 months visit and the answers were dichotomous (i.e., yes/no). Questions were categorized into four groups for analysis: 1) Play activities (10 questions), 2) Variety of play materials (8 questions), 3) Sources of play materials (3 questions), and 4) Household books (1 question). The sum of the ‘yeses’ were totaled for each of the four groups.
